# Current Trends and Perspectives in the Immune Therapy for Substance Use Disorders

**DOI:** 10.3389/fpsyt.2022.882491

**Published:** 2022-04-28

**Authors:** Octavian Vasiliu

**Affiliations:** Department of Psychiatry, Dr. Carol Davila University Emergency Central Military Hospital, Bucharest, Romania

**Keywords:** substance use disorders, vaccines, immune therapy, cocaine use disorder, nicotine use disorder

## Abstract

Substance use disorders (SUDs) are an extremely challenging category of disorders because of the high rate of relapse, lower life expectancy, important rate of psychiatric and somatic co-morbidity, lack of patients' insight during most of the disease duration, healthcare costs, etc. One of the reasons to consider these disorders very difficult for physicians and the healthcare system is the lack of adequate pharmacological agents with long-term proven efficacy. So far, there are no Food and Drug Administration (FDA) or European Medicines Agency (EMA)-approved treatments for most of the SUDs, except for alcohol use disorder, nicotine use disorder, and opioid use disorder. Immunotherapy has been considered a possible solution to SUDs because it may selectively target a certain drug of abuse, it may have a long-lasting effect (several weeks or months), and it ensures an adequate therapeutic adherence. The objective of this paper was to establish the current stage of research in the field of SUDs vaccines, based on a brief literature review. Vaccines for cocaine and nicotine dependence have reached phase III trials, while other researchers are focusing on passive immunization therapy for methamphetamine use disorder. New generations of vaccines are currently explored, and they are based on superior technologies compared to the first generation of immune therapy (e.g., viral transfer genes, more immunogenic adjuvants, or higher specificity haptens). Therefore, finding immune therapies for substance use disorders SUDs remains a matter of interest, and this approach may be useful for the management of an extremely dangerous and versatile psychiatric pathology.

## Introduction

Substance use disorders (SUDs) represent a complex and polymorphic pathology with severe psychological, social, and biological negative consequences (1). SUDs are responsible for significant rates of worldwide morbidity, mortality, quality of life impairments, financial and social burden, thus representing a major challenge for patients, their physicians, and caregivers, but also for the society and health care systems (2, 3). Despite SUDs' importance, there are currently only a few U.S. Food and Drug Administration (FDA) or European Medicines Agency (EMA)-approved medications, and for only a limited number of addictions, i.e., alcohol, nicotine, and opioid dependence (2, 3).

In the context of the COVID-19 pandemic, the problem of SUDs received increased attention, since recreational drug use may be perceived as a harmless way to cope with lockdown or isolation stress, and not as a gateway to addiction (4). Psychosocial stressors are well-known risk factors for SUDs, therefore search for new methods to cope with the challenge of substance abuse received a new impulse in the pandemic context (4, 5).

SUDs are frequently associated with psychiatric disorders or somatic diseases comorbidity, a phenomenon that lower life expectancy, alters patients' adherence to other, prescribed psychotropic or somatic treatments, increases healthcare costs, and decreases the probability of a complete functional recovery (6–8). Because there is a limited choice of drugs available for the treatment of SUDs, and these options are often criticized for the high rates of relapse, new strategies are acutely needed for these patients (9). The need to re-configure SUDs therapeutic management using a long-term approach, and not treatments focused only on the acute episodes of substance abuse, is supported by evidence like patients' lack of insight, high rate of treatment discontinuation, high risk of complications, and personality factors that are maintaining the addictive patterns (9–11). Therefore, a change of paradigm from orally-administered medicines to long-acting injectable treatments may be beneficial for patients, and the use of intramuscular injectable extended-release naltrexone is the first step in this direction (10, 12). Unlike oral naltrexone, the injectable formula does not imply first-pass metabolism in the liver, which allows for lower doses administration and lower peak plasma concentrations (10, 12).

Immunotherapy is another way to conceive a long-term treatment for SUDs with a focus on increased adherence and reduced risk for relapse. Another advantage of immunotherapy is targeting selective drugs of abuse, e.g., heroin, which is different from the non-selectivity of currently available, orally administered μ-opioid receptor (MOR) antagonists, for example (13). Unlike orally administered MOR antagonists, SUDs vaccines do not imply the need for prior detoxification, nor do they require daily supervision of treatment adherence.

The development of a vaccine, based on a hapten structurally similar to the target drug, which is conjugated to an immunogenic carrier protein, is considered able to elicit serum IgG antibodies and was associated with positive results in preclinical models of addiction (e.g., decreased drug self-administration, and attenuated the reward effects) (14). These antibodies are expected to sequester the target drug in order to prevent its entry into the brain, acting as an antagonist for the circulating drugs of abuse (14).

Several challenges are difficult to overcome, although significant progress has been made in this domain: lack of protection against a structurally dissimilar drug with the same pharmacodynamic properties as the drug of choice, lack of a significant effect over craving, which is responsible for relapse, and significant variability in antibody formation and their duration of life within blood circulation (15). The nature of haptens and adjuvants is very important in order to obtain a sustained and efficient response after the vaccine administration, in patients with SUDs. Despite the failure of first-generation conjugate vaccines against cocaine and nicotine in clinical trials, second-generation vaccines have shown superior results in preclinical models (14).

Regarding *the general benefits* of vaccines for SUDs, it is important to mention that this approach can offer the possibility of much closer treatment adherence monitoring (because the injectable treatment is administered only by a health care specialist, and it is not self-administered as the oral treatment), there are virtually no pharmacokinetic interactions, and these vaccines may significantly decrease the risk of overdose. *The limitations* are related to the variable titer of anti-bodies produced after vaccines administration, different selectivity and affinity of these products, and to the need to accept an injectable treatment by patients with low motivation for any type of therapy.

Combining multiple types of immune therapy, like an association of anti-drug antibodies with synthetic enzymes -that are able to stimulate abused substances metabolism- has also been investigated in preclinical models of SUDs (16). The purpose of this strategy was to explore the possible complementary action of anti-cocaine antibodies when added to cocaine hydroxylase (CoH) to decrease the drug uptake in the brain and to block the centrally-evoked locomotor stimulation (16). Synergistic actions of these types of interventions warrant further exploration in the treatment of selected SUDs (16). These methods involve viral gene transfer for a specific enzyme, e.g., CoH derived from human butyrylcholinesterase with the help of an adeno-associated viral vector, but these interventions are not yet approved for human trials (17).

## Objective

The primary objective of this review was to establish the stage of the current research in the field of SUD vaccines. The analysis of efficacy and tolerability of the investigational products targeting SUDs was based on a brief review of the clinical trials.

## Methodology

The search for investigational immunization products targeting SUDs included main clinical trials repositories run by the United States National Library of Medicine and the National Institutes of Health (clinicaltrials.gov) and the European Union (EU Clinical Trial Register). All phases of clinical investigation (I to IV) were considered for this review if trials were focused on specific SUDs and enrolled adult population.

In the second stage of this review, identifiers of the first stage-collected trials were included as keywords in the main electronic databases (PubMed, MEDLINE, Cochrane, Web of Science (Core Collection), PsychINFO, Scopus, and EMBASE in order to find associated, relevant articles containing study results. All *in-extenso* papers found in this stage were reviewed, and data about clinical trials methodology and results were synthesized in Table 1. If no published, relevant data were found in the second stage for specific trials, only methodological aspects were mentioned in Table 1. Data referring to animal studies were not included in the review.

**Table 1:** Active and passive immunization in SUDs.

**Anti-nicotine immunotherapy**					
**Nr**.	**CT identifier**	**I.P**.	**CT phase**	**Methods**	**POM and results**
1.	NCT00996034 (18)	NicVAX (3′AmNic-rEPA)	II	Occupancy of β2-nAChR by nicotine at baseline and following the administration of NicVAX 4–400 μg, using [123I]-5-IA-85380 SPECT; *N =* 11 healthy smokers; open-label	POM: mean of the average nicotine binding at scan 1 and 2 (baseline and 3 months) Results: Nicotine binding to β2-nAChR correlated positively with nicotine injected before but not after vaccination. The daily number of cigarettes and desire for a cigarette decreased after vaccination.
2.	NCT00598325 NCT00318383 (19)	NicVAX	II	Efficacy of NicVAX 200 and 400 μg 4–5 times over 6 months vs. placebo, *N =* 301 healthy smokers, DBRCT	POM: Anti-nicotine antibody concentration between screening and week 20, and from week 19 to 26, respectively Results: Vaccine recipients with the highest serum antinicotine antibodies level (top 30% AUC) were significantly more likely to attain 8 weeks of continuous abstinence (weeks 19–26) vs. placebo
3.	NCT00369616 (20)	NIC002	II	Efficacy and tolerability of NIC002 5 i.m injections vs. placebo, *N =* 341 smokers, DBRCT	POM: Abstinence rate (self-reported and measured by CO in exhaled air), immunogenicity (IgG antibodies measured by ELISA), safety and tolerability Results: The vaccine was safe, well-tolerated, and highly immunogenic after the first injection. The abstinence rate at month 2 was significant in favor of the vaccine., but continuous abstinence between months 2 and 6 was not significantly different. At 12 months, the difference in continuous abstinence rate between I.P. and placebo favored the I.P. only in those with high antibody response.
4.	NCT01304810 (21)	NicVAX	III	*N =* 300 participants who received 6 injections of NicVAX in previous trials, phase III, DBRCT, follow-up study, observational	POM: nicotine antibody levels 24 months after injection Results: undisclosed
5.	NCT01318668, EudraCT Number: 2010-019381-90 (22)	NicVAX	I/II	Effects of NicVAX 400 μg vs. placebo over CNS activation and behavior following a nicotine challenge, using fMRI, *N =* 48 participants, smokers of ≥10 cigarettes per day, DBRCT	POM: fMRI at 18 and 20 weeks post-vaccination, and reaction time in a battery of psychomotor tests Results: No difference in brain activity to smoking cues between treatment groups; no effects of acute nicotine challenge were observed, either.
6.	NCT01102114 (23)	NicVAX	III	Efficacy, immunogenicity, and safety of NicVAX as an aid to smoking cessation, *N =* 1,000 healthy smokers, 6 doses over 6 months, DBRCT	POM: Efficacy of NicVAX in reaching abstinence (by self-report and CO confirmation) during 12 months Results: undisclosed
7.	NCT01178346 (24)	NicVAX	III	Pharmacoeconomic of NicVAX vs. placebo, *N =* 500, non-randomized	POM: Health-related QoL changes during NicVAX administration – one-year monitoring Results: undisclosed
8.	NCT01672645 (25)	NIC7-001, NIC7-003	I	Safety and tolerability of NIC7-001/003 vs. placebo, *N =* 277 healthy smokers, DBRCT	POM: Adverse events (local and systemic) Results: undisclosed
9.	NCT01478893 (26)	SEL-068	I	Safety and pharmacodynamics of SEL-068 vs. placebo, DBRCT, *N =* 82 healthy smokers	POM: Frequency and severity of adverse events during 36 weeks Results: undisclosed
10.	NCT00836199 (27)	NicVAX	III	Efficacy, immunogenicity, and safety of NicVAX vs. placebo, *N =* 1,000, 6 doses over 6 months, DBRCT	POM: One-year abstinence rate under NicVAX as an aid to smoking cessation Results: undisclosed
11.	NCT00218413 (28)	NicVAX	II	Safety and immunogenicity NicVAX 100, 200, 300, or 400 μg, *N =* 51 smokers, open-label	POM: Antinicotineantibody concentrations from baseline to day 365 Results: undisclosed
12.	NCT00995033, EudraCT Number: 2008-005894-36 (29)	NicVAX, varenicline	IIb	Efficacy and safety of NicVAX/placebo + varenicline, *N =* 558 healthy smokers	POM: Long term abstinence (1 year) Results: undisclosed
13.	NCT01280968 (30)	NIC002 (NicQBeta) + Aluminum hydroxide vs. placebo	II	Efficacy of NIC002 100 μg 4 injections over 3 months vs. placebo, *N =* 52 smokers, DBRCT	POM: Vaccine induces percent change in brain nicotine AUC/Cmax/T1/2/initial slope of brain nicotine accumulation after a single/multiple puffs Results: submitted, but yet unpublished
14.	NCT00736047, EudraCT Number: 2007-006741-40 (31)	NIC002	II	Efficacy, safety, tolerability, and immunogenicity of NIC002 vs. placebo, *N =* 200 smokers, DBRCT	POM: Smoking status, exhaled CO (12 months) Results: disclosed, but unpublished
15.	NCT00633321, EudraCT Number: 2005-000922-22 (32)	TA-NIC	II	Efficacy and safety of TA-NIC 100 or 250 μg vs. placebo, *N =* 522 smokers, DBRCT	POM: Smoking quit rate of minimum 4 weeks determined at week 26 (self-report and CO breath test data) Results: submitted, but yet unpublished
**Immunotherapy for cocaine use disorder**					
16.	NCT00965263 (33)	TA-CD	II	Evaluation of the relation between antibody titers and the effects of smoked cocaine on rates of intoxication, craving, and cardiovascular effects, TA-CD 82 or 360 μg administered 4 times, *N =* 10, DBRCT	POM: Cocaine intoxication during 13 weeks (effect evaluated by VAS) Results: Peak plasma antibody levels significantly predicted cocaine's effects. Patients with higher titers of antibodies had an immediate and robust reduction in ratings of VAS, while those in the inferior half showed only non-significant attenuation. Self-reported use of cocaine tended to decrease as a function of antibody titer. Higher antibody titer predicted significantly greater cocaine-induced tachycardia.
17.	NCT00969878, EudraCT Number: 2008-002183-34 (34)	TA-CD	II	Efficacy of TA-CD 82 or 360 μg administered 4 times vs. placebo, *N =* 300 patients with CUD, DBRCT	POM: Rate of at least 2 weeks cocaine abstinence during weeks 9 to 16 (cocaine-free urines) Results: Almost 3-times fewer high-level anti-cocaine IgG subjects dropped out compared to low-titers subjects. No difference between the three study groups was detected by the POM for the full 16 weeks of the trial. After week 8 more vaccinated than placebo subjects attained abstinence for ≥2 weeks, but not significant. No treatment-related SAE withdrawal was reported.
18.	NCT00142857 (35)	TA-CD	IIb	Efficacy of TA-CD 360 μg administered 5 times vs. placebo, *N =* 115 CUD patients maintained on methadone, DBRCT	POM: At least 2 weeks cocaine abstinence during weeks 9 to 16 after vaccination Results: Subjects reaching high levels of serum IgG anti-cocaine antibodies (≥43 μg/ml) had significantly more cocaine-free urine samples than those with low levels and those receiving placebo, during weeks 9 to 16. Subjects with a 50% reduction in cocaine use were significantly more in the high IgG titers group vs. low IgG levels. No SAE related to treatment was reported.
19.	NCT02455479 (36)	dAd5GNE	I	Safety and preliminary efficacy of the vaccine vs. placebo, *N =* 30 (estimated) CUD patients, DBRCT	POM: General and specific safety parameters Results: the trial is ongoing as of February 2022
**Immunotherapy for methamphetamine use disorder**					
20.	NCT01603147 (37)	ch-mAb7F9	I	Safety of the I.P. vs. placebo, *N =* 42 healthy volunteers, DBRCT	POM: Adverse events, vital signs, ECG, clinical laboratory testing over 21 weeks Results: undisclosed
21.	NCT05027451 (38)	IXT-m200	I	Safety, tolerability, and pharmacokinetics of a 3 g single-dose i.v.- administered I.P. vs. placebo, *N =* 9 healthy subjects, DBRCT	POM: Treatment-related AE assessed by physical examination, ECG, laboratory testing, and vital signs during 127 days Results: undisclosed
22.	NCT03336866 (39)	IXT-m200	I/II	Efficacy of I.P (6 or 20 mg/kg i.v. dose) to change methamphetamine concentrations in blood and to alter methamphetamine feels vs. placebo, *N =* 56 MUD, DBRCT	POM: Change in plasma methamphetamine AUC or Cmax after challenges following single i.v doses of I.P. (29 days) Results: submitted, not yet published

*CT, clinical trial; I.P., investigational product; POM, primary outcome measures; DBRCT, double blind randomized clinical trial; β2-nAChR, β2-containing nicotinic acetylcholine receptors; SPECT, single photon emission computed tomography; CNS, central nervous system; AUC, area under curve; QoL, quality of life; i.m, intramuscular; i.v, intravenous; VAS, Visual Analog Scale; CUD, cocaine use disorder; SAE, serious adverse event; ECG, electrocardiogram; AUC, area under the curve; MUD, methamphetamine use disorder*.

## Results

A number of 22 phase I to III clinical trials referring to nicotine use disorder, cocaine use disorder, and methamphetamine use disorder, including both active and passive immunization products, were found after the primary search. Out of these 22 trials, only 7 have been identified as having published results in the secondary search. The vast majority of identified trials were phase II, targeting nicotine use disorder, using active immunization (NicVAX, NIC002, TA-NIC, NIC7-001/003, and SEL-068), and presented mixed results (18–20). The tolerability of these vaccines was good, but continuous abstinence was significant only in subjects with the highest serum antinicotine levels (19), or it was significant only in short term (20). No published results of phase III trials evaluating vaccines for nicotine use disorder were found (21–24).

Immunization for patients with cocaine use disorder (TA-CD, dAd5GNE) was explored in phase I and II clinical trials (33–36). Self-reported use of cocaine tended to decrease as a function of antibody titer in one trial (33), while in another trial subjects reaching high levels of serum IgG anti-cocaine antibodies (≥43 μg/ml) had significantly more cocaine-free urine samples than those with low levels and those receiving placebo, during week 9–16 (35). Although yet another trial with TA-CD did not reach its primary outcome after week 8 more vaccinated than placebo subjects attained abstinence for ≥2 weeks, without reaching the significance level (34).

Immunotherapy for methamphetamine use disorder is still in its early phase, and passive immunization is the only clinically explored option (37–39). This therapy is based on human-murine chimeric monoclonal antibodies, which are considered able to bind methamphetamine with presumed high specificity and affinity (37–39). Unfortunately, no results of clinical trials are yet available to support this claim.

The evolution of the research in the field of immunotherapy for SUD is reflected by the development of second-generation investigational products. This progress was fueled by the need to find newer haptens and better carriers, able to induce more specific and intense immune responses, which translate to better efficacy. Also, adjuvants are important for triggering a persistent immune response, and new substances from this category are needed, besides aluminum.

In the case of anti-nicotine vaccines, the first generation of products used 3'-aminomethyl nicotine conjugated with Pseudomonas aeruginosa r-exoprotein in case of NicVAX, a non-infectious pseudo-viral particle (VLP) in case of NIC-002, or recombinant cholera toxin B (rCTB) subunit as transporter molecule for TA-NIC (18, 20). In the case of second-generation products, the transporter is represented by cross-reacting material (CRM) for NIC7-001, and a nanoparticle technology (targeted synthetic particles) for SEL-068 (25, 26). In the case of cocaine use disorder, the first-generation vaccine, TA-CD, used rCTB, while the newest investigational product, dAd5GNE, used the proteins of a modified adenovirus conjugated with a cocaine analog (33–36).

An overview of the trials presented in Table 1 shows the need to find better defined primary outcome measures and to extend the duration of monitoring over 12 months. Also, trials with active comparators should be designed, and quantification of the patients' tolerability and quality of life during the study could also be useful.

## Conclusions

The limited number of phase III clinical trials detected by this review (*N* = 4) indicates the need to re-evaluate the conceptual design of immune therapy in SUDs (Table 2). Despite the mixed, and mostly negative evidence of efficacy for the first-generation conjugate vaccines against cocaine and nicotine in clinical trials, second-generation vaccines are developing, thus renewing the potential clinical utility of active immunization in the treatment of substance use disorder (14). Even the first generation trials contained several positive results, but only in specific sub-populations, i.e., patients able to develop high levels of antibodies targeting the specific drug of abuse (19, 35). No significant tolerability and safety aspects were reported in trials with published results, which monitored the adverse events rate.

**Table 2:** Synthetic presentation of clinical trials for immune therapy in SUDs.

**Phase I**	**Phase II**	**Phase III**	**Phase IV**
NUD *N =* 3	NUD = 9	NUD = 4	-
CUD = 1	CUD = 3	-	-
MUD = 3	MUD = 1	-	-

*NUD, nicotine use disorder; CUD, cocaine use disorder; MUD, methamphetamine use disorder*.

As limitations of the review, it is important to mention that no data about the current status of each investigational product were collected, as no such information has been found in the searched databases. Note releases from the manufacturers' sites regarding these products were not included in this review. Preclinical studies may be important to review, but this article was focused only on human trials.

## Author Contributions

The author confirms being the sole contributor of this work and has approved it for publication.

## Conflict of Interest

The author declares that the research was conducted in the absence of any commercial or financial relationships that could be construed as a potential conflict of interest.

## Publisher's Note

All claims expressed in this article are solely those of the authors and do not necessarily represent those of their affiliated organizations, or those of the publisher, the editors and the reviewers. Any product that may be evaluated in this article, or claim that may be made by its manufacturer, is not guaranteed or endorsed by the publisher.

## References

[1] Daley DC. Family and social aspects of substance use disorders and treatment. J Food Drug Anal. (2013) 21:S73–S76. 10.1016/j.jfda.2013.09.038 25214748 PMC4158844

[2] Volkow NA. Personalizing the treatment of substance use disorders. Am J Psychiatry. (2020) 177:113–6. 10.1176/appi.ajp.2019.19121284 32008390

[3] Connery HS McHugh RK Reilly M Shin S Greenfield SF. Substance use disorders in global mental health delivery: epidemiology, treatment gap, and implementation of evidence-based treatments. Harv Rev Psychiatry. (2020) 28:316–27. 10.1097/HRP.0000000000000271 32925514 PMC8324330

[4] Vasiliu O Vasile D Vasiliu DG Ciobanu OM. Quality of life impairments and stress coping strategies during the Covid-19 pandemic isolation and quarantine- A web-based survey. Rom J Mil Med. (2021) 124:10–21. 10.55453/rjmm.2021.124.1.2

[5] López-Pelayo H Aubin HJ Drummond C Dom G Pascual F Rehm J. “The post-COVID era”: challenges in the treatment of substance use disorder (SUD) after the pandemic. BMC Med. (2020) 18:241. 10.1186/s12916-020-01693-9 32731868 PMC7392642

[6] Schuckit MA. Comorbidity between substance use disorders and psychiatric conditions. Addiction. (2006) 101:76–88. 10.1111/j.1360-0443.2006.01592.x 16930163

[7] Langås AM Malt UF Opjordsmoen S. Comorbid mental disorders in substance users from a single catchment area- a clinical study. BMC Psychiatry. (2011) 11:25. 10.1186/1471-244X-11-25 21314980 PMC3042931

[8] Onyeka IN Høegh MC Eien EMN Nwaru BI Melle I. Comorbidity of physical disorders among patients with severe mental illness with and without substance use disorders: a systematic review and meta-analysis. J Dual Diagn. (2019) 15:192–206. 10.1080/15504263.2019.1619007 31164045

[9] McLellan AT Lewis DC O'Brien CP Kleber HD. Drug dependence, a chronic medical illness: Implications for treatment, insurance, and outcomes evaluation. J Am Med Assoc. (2000) 284:1689–95. 10.1001/jama.284.13.1689 11015800

[10] Vasiliu O. Maintenance pharmacologic therapies for opioid use disorders: beyond opioid agonists. Rom J Mil Med. (2019) CXXII:52–70. 27391852

[11] Vasiliu O. Current status of evidence for a new diagnosis: food addiction- A literature review. Front Psychiatry. (2022) 12:824936. 10.3389/fpsyt.2021.824936 35082706 PMC8784968

[12] Jarvis BP Holtyn AF Subramaniam S Tompkins DA Oga EA Bigelow GE. Extended-release injectable naltrexone for opioid use disorder: a systematic review. Addiction. (2018) 113:1188–209. 10.1111/add.14180 29396985 PMC5993595

[13] Niciu MJ Arias AJ. Targeted opioid receptor antagonists in the treatment of alcohol use disorders. CNS Drugs. (2013) 27:777–87. 10.1007/s40263-013-0096-4 23881605 PMC4600601

[14] Bremer PT Janda KD. Conjugate vaccine immunotherapy for substance use disorder. Pharmacol Rev. (2017) 69:298–315. 10.1124/pr.117.013904 28634286 PMC5482184

[15] Kantak KM. Vaccines against drugs of abuse: a viable treatment option? Drugs. (2003) 63:341–52. 10.2165/00003495-200363040-00001 12558457

[16] Brimijoin S Orson F Kosten T Kinsey B Shen XY White SJ. Anti-cocaine antibody and butyrylcholinesterase-derived cocaine hydrolase exert cooperative effects on cocaine pharmacokinetics and cocaine-induced locomotor activity in mice. Chem Biol Interact. (2013) 203:212–16. 10.1016/j.cbi.2012.08.015 22960160 PMC3572300

[17] Gao Y Geng L Orson F Kinsey B Kosten TR Shen X. Effects of anti-cocaine vaccine and viral gene transfer of cocaine hydrolase in mice on cocaine toxicity including motor strength and liver damage. Chem Biol Interact. (2013) 203:208–11. 10.1016/j.cbi.2012.08.006 22935511 PMC3537841

[18] Esterlis I Hannestad JO Perkins E Bois F D'Souza DC Tyndale RF. Effect of a nicotine vaccine on nicotine binding to β2^*^-nicotinic acetylcholine receptors in vivo in human tobacco smokers. Am J Psychiatry. (2013) 170:399–407. 10.1176/appi.ajp.2012.12060793 23429725 PMC3738000

[19] Hatsukami DK Jorenby DE Gonzales D Rigotti NA Glover ED Oncken CA. Immunogenicity and smoking-cessation outcomes for a novel nicotine immunotherapeutic. Clin Pharmacol Ther. (2011) 89:392–9. 10.1038/clpt.2010.317 21270788 PMC4106715

[20] Cornuz J Zwahlen S Jungi WF Osterwalder J Klinger K van Melle G. A vaccine against nicotine for smoking cessation: a randomized controlled trial. PLoS ONE. (2008) 3:e2547. 10.1371/journal.pone.0002547 18575629 PMC2432028

[21] Nabi Pharmaceuticals. A follow-up study to assess long-term immunogenicity and safety of NicVAX/Placebo as an aid to smoking cessation. Available online at: https://www.clinicaltrials.gov/ct2/show/NCT01304810 (accessed: February 10, 2022).

[22] Havermans A Vuurman EF van der Hurk Hoogsteder P van Schayck OCP. Treatment with a nicotine vaccine does not lead to changes in brain activity during smoking cue exposure or a working memory task. Addiction. (2014) 109:1260–7. 10.1111/add.12577 24894701

[23] Nabi, Pharmaceuticals. A Second Study of NicVAX/Placebo as an Aid for Smoking Cessation. Available online at: https://clinicaltrials.gov/ct2/show/NCT01102114 (accessed: February 12, 2022).

[24] Nabi, Pharmaceuticals. Pharmacoeconomic Assessment in Nabi-4514 and Nabi-4515 Phase 3 Studies. Available online at: https://clinicaltrials.gov/ct2/show/NCT01178346 (accessed: February 12, 2022).

[25] Pfizer. A Study to Assess the Safety and Tolerability of Different Doses of PF-05402536 and PF-06413367 in Healthy Adult Smokers. Available online at: https://clinicaltrials.gov/ct2/show/NCT01672645 (accessed: February 20, 2022).

[26] Selecta, Biosciences, Inc. Safety and Pharmacodynamics of SEL_068 Vaccine in Smokers and Non-Smokers. Available online at: https://clinicaltrials.gov/ct2/show/NCT01478893 (accessed: February 20, 2022).

[27] Nabi, Pharmaceuticals. NicVAX/placebo as an Aid for Smoking Cessation. Available online at: https://clinicaltrials.gov/ct2/show/NCT00836199. (accessed: February 12, 2022).

[28] Nabi, Pharmaceuticals. Safety and Effectiveness of NicVAX In Treating Nicotine-Dependent Individuals. Available online at: https://clinicaltrials.gov/ct2/show/NCT00218413 (accessed: February 12, 2022).

[29] Maastricht University Medical Center. Efficacy and Safety of NicVAX co-Administered With Varenicline (Champix). Available online at https://clinicaltrials.gov/ct2/show/NCT00995033. (accessed: February 16, 2022).

[30] Alexey, Mukhin. Improving the Efficacy of Anti-Nicotine Immunotherapy (PETNic002). Available online at: https://clinicaltrials.gov/ct2/show/NCT01280968 (accessed: February 16, 2022).

[31] Novartis. Study to Evaluate the Efficacy, Safety, Tolerability and Immunogenicity of 100 μ*g NIC002 Vaccine in Cigarette Smokers Who Are Motivated to Quit Smoking*. Available online at: https://clinicaltrials.gov/ct2/show/NCT00736047 (accessed: February 16, 2022).

[32] Celtic, Pharma Development Sevices. Study of TA-NIC to Assess the Efficacy and Safety of the Vaccine as an Aid to Smoking Cessation. Available online at: https://clinicaltrials.gov/ct2/show/results/NCT0063332 (accessed: February 20, 2022).

[33] Haney M Gunderson EW Jiang H Collins ED Foltin RW. Cocaine-specific antibodies blunt the subjective effects of smoked cocaine in humans. Biol Psychiatry. (2010) 67:59–65. 10.1016/j.biopsych.2009.08.031 19846066 PMC3319755

[34] Kosten TR Domingo CB Shorter D Orson F Green C Somoza E. Vaccine for cocaine dependence: a randomized double-blind placebo-controlled efficacy trial. Drug Alcohol Depend. (2014) 140:42–7. 10.1016/j.drugalcdep.2014.04.003 24793366 PMC4073297

[35] Martell BA Orson FM Poling J Mitchell E Rossen RD Gardner T. Cocaine vaccine for the treatment of cocaine dependence in methadone-maintained patients: a randomized, double-blind, placebo-controlled efficacy trial. Arch Gen Psychiatry. (2009) 66:1116–23. 10.1001/archgenpsychiatry.2009.128 19805702 PMC2878137

[36] Weill Medical College of Cornell University. Safety study of a disrupted adenovirus (Ad) serotype cocaine vaccine for cocaine-dependent individuals. Available online at: https://clinicaltrials.gov/ct2/show/NCT02455479 (accessed: February 20, 2022).

[37] InterveXion, Therapeutics. Safety Study of Ch-mAb7F9 for Methamphetamine Abuse. Available online at: https://clinicaltrials.gov/ct2/show/NCT01603147 (accessed: February 20, 2022).

[38] InterVexion, Therapeutics. Safety, Tolerability, and Pharmacokinetics of IXT-m200. Available online at: https://clinicaltrials.gov/ct2/show/NCT05027451 (accessed: February 20, 2022).

[39] InterveXion, Therapeutics. Study of Antibody for Methamphetamine Outpatient Therapy (STAMPOUT). Available online at: https://clinicaltrials.gov/ct2/show/NCT03336866 (accessed: February 20, 2022).

